# On the use of a convolution–superposition algorithm for plan checking in lung stereotactic body radiation therapy

**DOI:** 10.1120/jacmp.v17i5.6186

**Published:** 2016-09-08

**Authors:** Nicholas Hardcastle, Bradley M. Oborn, Annette Haworth

**Affiliations:** ^1^ Northern Sydney Cancer Centre, Royal North Shore Hospital St. Leonards NSW; ^2^ Centre for Medical Radiation Physics, University of Wollongong Wollongong NSW; ^3^ Illawarra Cancer Care Centre Wollongong NSW; ^4^ Department of Physical Sciences Peter MacCallum Cancer Centre East Melbourne VIC; ^5^ The Sir Peter MacCallum Department of Oncology University of Melbourne, Melbourne Victoria Australia

**Keywords:** lung, SBRT, SABR, convolution–superposition, Mobius, Monte Carlo

## Abstract

Stereotactic body radiation therapy (SBRT) aims to deliver a highly conformal ablative dose to a small target. Dosimetric verification of SBRT for lung tumors presents a challenge due to heterogeneities, moving targets, and small fields. Recent software (M3D) designed for dosimetric verification of lung SBRT treatment plans using an advanced convolution–superposition algorithm was evaluated. Ten lung SBRT patients covering a range of tumor volumes were selected. 3D CRT plans were created using the XiO treatment planning system (TPS) with the superposition algorithm. Dose was recalculated in the Eclipse TPS using the AAA algorithm, M3D verification software using the collapsed‐cone‐convolution algorithm, and in‐house Monte Carlo (MC). Target point doses were calculated with RadCalc software. Near‐maximum, median, and near‐minimum target doses, conformity indices, and lung doses were compared with MC as the reference calculation. M3D 3D gamma passing rates were compared with the XiO and Eclipse. Wilcoxon signed‐rank test was used to compare each calculation method with XiO with a threshold of significance of p<0.05. M3D and RadCalc point dose calculations were greater than MC by up to 7.7% and 13.1%, respectively, with M3D being statistically significant (s.s.). AAA and XiO calculated point doses were less than MC by 11.3% and 5.2%, respectively (AAA s.s.). Median and near‐minimum and near‐maximum target doses were less than MC when calculated with AAA and XiO (all s.s.). Near‐maximum and median target doses were higher with M3D compared with MC (s.s.), but there was no difference in near‐minimum M3D doses compared with MC. M3D‐calculated ipsilateral lung V20 Gy and V5 Gy were greater than that calculated with MC (s.s.); AAA‐ and XiO‐calculated V20 Gy was lower than that calculated with MC, but not statistically different to MC for V5 Gy. Nine of the 10 plans achieved M3D gamma passing rates greater than 95% and 80%for 5%/1 mm and 3%/1 mm criteria, respectively. M3D typically calculated a higher target and lung dose than MC for lung SBRT plans. The results show a range of calculated doses with different algorithms and suggest that M3D is in closer agreement with Monte Carlo, thus discrepancies between the TPS and M3D software will be observed for lung SBRT plans. M3D provides a useful supplement to verification of lung SBRT plans by direct measurement, which typically excludes patient specific heterogeneities.

PACS number(s): 87.55.D‐, 87.55.Qr, 87.55.K‐

## I. INTRODUCTION

Stereotactic body radiation therapy (SBRT) involves the application of highly conformal dose distributions to small primary tumors and oligometastases with the aim of ablating local disease.^1^, ^2^ In the lung, SBRT presents a challenge for dose calculation algorithms due to the small fields and high degrees of density heterogeneity leading to lateral electronic disequilibrium.^3^, ^4^ Due to the increasing role of lung SBRT and the potential for unintended consequences if the dose is not calculated correctly, plan verification for lung SBRT must be both efficient and accurate.

Use of plan verification software that applies simplistic Type A dose calculation algorithms (those that do not take into account lateral scatter variations with density),^(5)^ may lead to dose discrepancies in lung SBRT plans that are difficult to resolve. Validation by measurement of SBRT plans is frequently performed using a high‐resolution detector such as film in a homogeneous media which will detect dose delivery or small field modeling issues. A measurement approach, however, can be inefficient and misses one of the largest sources of uncertainty — calculation of dose in a small dense volume surrounded by low density lung tissue. Due to the large range in tumor sizes and their variable location within the lung and/or proximity to critical structures such as the esophagus or heart, a standard phantom cannot recreate the exact conditions of the plan to be checked.

Whilst the accuracy of the majority of commercial treatment planning systems is well documented under standard planning conditions, extrapolating these known limitations to the demands of lung SBRT treatments is not straightforward. Furthermore, all dose calculation algorithms incorporate varying levels of approximation to produce plans in desired time frames. Hence, an independent dose calculation algorithm that takes into account changes in lateral scatter conditions is highly desirable to understand uncertainties in dose calculation algorithms used for clinical treatment planning.

This study evaluates dose calculation in Mobius3D (M3D, Mobius Medical Systems, Houston, TX) in the context of lung SBRT via comparison with a Monte Carlo dose calculation model. We then compare this performance with that of two commonly used commercial treatment planning systems and a Type A‐based plan verification software to determine the suitability of this software for calculation‐based plan verification. The M3D software is implemented using a graphical processing unit which allows fast computation of dose distributions, reducing the need for compromise in dose computation accuracy.^(6)^ This work builds on previous publications that have demonstrated the performance of the M3D software for independent plan verification of typical IMRT and VMAT fields that are more easily benchmarked against measurement in homogeneous media.^7^, ^8^, ^9^

## II. MATERIALS AND METHODS

Ten lung SBRT plans with target volumes 0.7 cm^3^ to 20 cm^3^ were selected representing the range of typical lesion sizes in our institution. Moreover, a range of tumor locations was selected which include medial, on chest wall, and central within the lung. The plans were calculated with two treatment planning systems utilizing different Type B dose calculation algorithms. Secondary plan checks were performed with two different plan check software, one of which employs a collapsed cone convolution dose calculation algorithm (M3D), whereas the other employs a correction‐based algorithm similar to a Type A dose calculation algorithm. A final calculation of each plan was also performed with an in‐house Monte Carlo plan verification system, which has previously demonstrated accurate dose calculation in heterogeneous media. The Monte Carlo calculation was used as the comparison dataset, as it was assumed this was the most accurate dose calculation algorithm of those evaluated. Verifying the dose by direct measurement in a standard (homogeneous) phantom would not assess the performance of any of the systems in the presence of patient‐specific, small, dense target volumes surrounded by low‐density lung tissue and therefore was not included in this study.

Treatment planning scans were created using average intensity projections of 4D CTs, on which the internal target volume (ITV) was contoured based on the maximum intensity projection of all phases. All calculations were performed for a Varian 21iX linear accelerator with 6 MV photons and a Millenium multileaf collimator (Varian Medical Systems, Palo Alto, CA). All collimator, gantry, patient density, and monitor unit settings were consistent between all calculations.

### A. Treatment planning system dose calculation

The clinical treatment plans were initially created using the XiO treatment planning system (v4.7, Elekta AB, Stockholm, Sweden). A 3D conformal approach utilizing eight to nine ipsilateral beams, including one to two noncoplanar beams, was used.^(10)^ Planning constraints are given in Table 1. The prescribed dose was 26 Gy in a single fraction such that 100% of the prescription dose covered 99% of the planning target volume (PTV), per our institutional guidelines. The maximum dose was typically of the order of 125% of the prescription dose. The superposition dose calculation algorithm was chosen with a dose calculation resolution of 2.5 mm anterior–posterior/left–right and 3 mm superior–inferior.^11^, ^12^ The superposition dose calculation utilizes a collapsed‐cone convolution dose calculation method that involves the convolution of a Total Energy Released per unit MAss (TERMA) volume with Monte Carlo‐calculated point energy deposition kernels. This algorithm allows for density scaling of the energy deposition kernels to account for local density heterogeneities.

The DICOM RT plan, CT and structure sets were imported into the Eclipse treatment planning system (V.11, Varian Medical Systems) where the dose was recalculated using the analytic anisotropic algorithm (AAA, v11.0.31), using a dose grid resolution of 2.5 mm in each direction. The AAA algorithm uses pencil beam energy deposition kernels generated from Monte Carlo. The kernels are represented analytically and include scaling based on local density variations, with specific analytic kernels used for buildup/down effects at density interfaces.^(13)^ Both treatment planning system calculations utilized the same CT to relative electron density curve and monitor units.

**Table 1:** Planning dose constraints.

*Organ*	*Constraint*
Heart	Maximum dose <15 Gy
Lung	D20%<20 Gy
Lung	D66%<5 Gy
Oesophagus	Maximum dose <15.4 Gy
Brachial plexus	Maximum dose <15.0 Gy
Chest wall	Maximum dose to 30 cc <24 Gy
Skin	Maximum dose <24.0 Gy
Spinal cord	Maximum dose <12.0 Gy
PTV	D99%>26 Gy
PTV	Maximum dose ≈ 32.5 Gy

### B. Plan verification

The DICOM RT plan, CT, dose, and structure sets were imported into M3D (v1.4, Mobius Medical Systems) from the XiO TPS. M3D uses a collapsed cone convolution–superposition algorithm that utilizes the convolution of a TERMA volume with Monte Carlo‐derived point spread kernels, implemented on a graphics processing unit (GPU).^(6)^ The 6 MV photon beam model in M3D was configured to match the dose at 10 cm in water for a $10\times 10\;{\text{cm}}^{2}$ field at 100 cm source to surface distance as part of the initial setup. It is recommended by the manufacturer that, unless the default beam data varies from the locally measured clinical data by greater than 3%, these values should not be changed. The maximum deviation observed between our beam data and the Mobius default data for the geometries relevant to SBRT (no wedges, less than 10 cm field size) was 2%, therefore the default values were not modified. Furthermore, the default CT to relative electron density curve provided with the system was utilized for all M3D calculations.

The DICOM RT plan, CT, point dose, and structure sets were also imported into the RadCalc (v6.2, Lifeline Software, Austin, TX) monitor unit check software and the dose to the calculation point in the center of the GTV in each plan was calculated. Field size scaling was employed for targets that were surrounded by lung tissue. This scales the output factor by the ratio of the actual and radiological path lengths, and is a simplistic correction applied to account for the lack of lateral scatter in tumors surrounded by lung.

### C. Monte Carlo dose calculation

An EGSnrc (BEAMnrc/DOSXYZnrc) based in‐house Monte Carlo system was used. This system has been benchmarked in previous work and is based on full absolute calibration style simulations.^(14)^ The 6 MV beam model used an electron spot‐size of 1.2 mm×0.9 mm, with a 6.5 MeV peak (0.1 MeV FHWM spread with no angular divergence). The treatment head was modeled using BEAMnrc, with ECUT and PCUT values of 0.521 MeV and 0.01 MeV, respectively. For the phantom calculations, the DOSXYZnrc module was used with ECUT=0.7 MeV and PCUT=0.01 MeV. Benchmarking simulations were performed by comparing the MC results to measurements performed in water with a CC13 ionization chamber. A 100% gamma passing rate was observed for 2%/3 mm criteria for central axis percentage depth‐dose profiles and cross‐profiles at 15 mm and 100 mm depth, for both $4\times 4\;{\text{cm}}^{2}$ and $10\times 10\;{\text{cm}}^{2}$ beams.

For all patient‐based calculations, the patient's CT data were converted to egsphant format files and read into the DOSXYZnrc module. The voxel sizes chosen corresponded to 2 pixels in the slice plane and 1 in the slice direction, that is, $2.14\times 2.14\times 3\;{\text{mm}}^{3}$. The materials simulated were air ($0.0013\;g/{\text{cm}}^{3}$) and water densities ranging from $0.085\;g/{\text{cm}}^{3}$ to 2 g/cm^3^ in bins of width $0.05\;g/{\text{cm}}^{3}$. The dose is, therefore, reported as dose to water, transported in water. This is designed to match the processes modeled by the treatment planning systems and M3D. Simulating more realistic grades of materials, such as bone and lung, would make direct comparison more complicated and is not the focus of the current work.

The DICOM RT plan was converted into BEAMnrc input files. For all simulations a total of $40\times 10^{9}$ primary histories were used to generate phase space files for each beam, scored just below the MLC plane. These phase space files were then input into the DOSXYZnrc simulations and recycled 24 times in order to reduce the voxel dose error estimates in the PTV to <2%.

### D. Comparison of doses

All computed doses were exported from their respective platforms and imported into Mim Maestro (v6.3, Mim Software, Clevelend, OH) such that comparison of the calculated doses was performed on an independent platform. Mim allows visualization of dose distributions, RT structures, and CT images, and provides a platform for comparison of point and volumetric dose metrics. A number of dosimetric parameters describing target and organs‐at‐risk (OAR) doses were compared between the calculation methods. Institutional protocols, clinical requirements, and ICRU 83 reporting recommendations determined the choice of metrics. The 100% and 50% isodose line conformity indices were calculated as the volume receiving 100% and 50% of the prescription dose divided by the PTV volume. These were compared between the algorithms as a measure of the dose falloff surrounding the target volume. The Wilcoxon signed‐rank test was used to compare each calculation algorithm with the Monte Carlo calculation, with a threshold for statistical significance of p<0.05.

The four calculation algorithms used for 3D calculation all have different linac and multileaf collimator modeling methods. To set a benchmark for comparison of the different algorithms independent of heterogeneity corrections, a single plan (Patient 6) was recomputed in each TPS, M3D, and MC, with the body contour set to a density of 1 g/cm^3^ and the couch contour set to a density of $0.25\;g/{\text{cm}}^{3}$ (according to the standard clinical couch correction protocol). The ICRU 83 metrics were compared between the algorithms, relative to the MC calculation.

RadCalc reports the point dose in the tumor and a threshold for agreement with the treatment planning system of 5% was used as per institutional guidelines. The output from the M3D software includes a number of parameters to aid plan assessment. To be consistent with previous publications reporting the performance of M3D with IMRT and VMAT plans, we include the 3D gamma analysis statistics for the SBRT plans. For these plans, a global gamma analysis was used to report the number of pixels passing at the 5%/1 mm and 3%/1 mm criteria with a low dose threshold of 10%. These gamma criteria were selected based on AAPM TG 40. It recommends a 5% agreement is expected when using substantial field blocking or heterogeneity corrections, a 1 mm agreement is expected when working in tight dose gradients around critical structures typically encountered in SBRT treatments, and 3% was applied to generate a ‘signal’ when plans easily pass the 5% criteria. RadCalc reports the point dose in the tumor, and a threshold for agreement with the treatment planning system of 5% was used as per institutional guidelines.

## III. RESULTS

**Figure 1:**
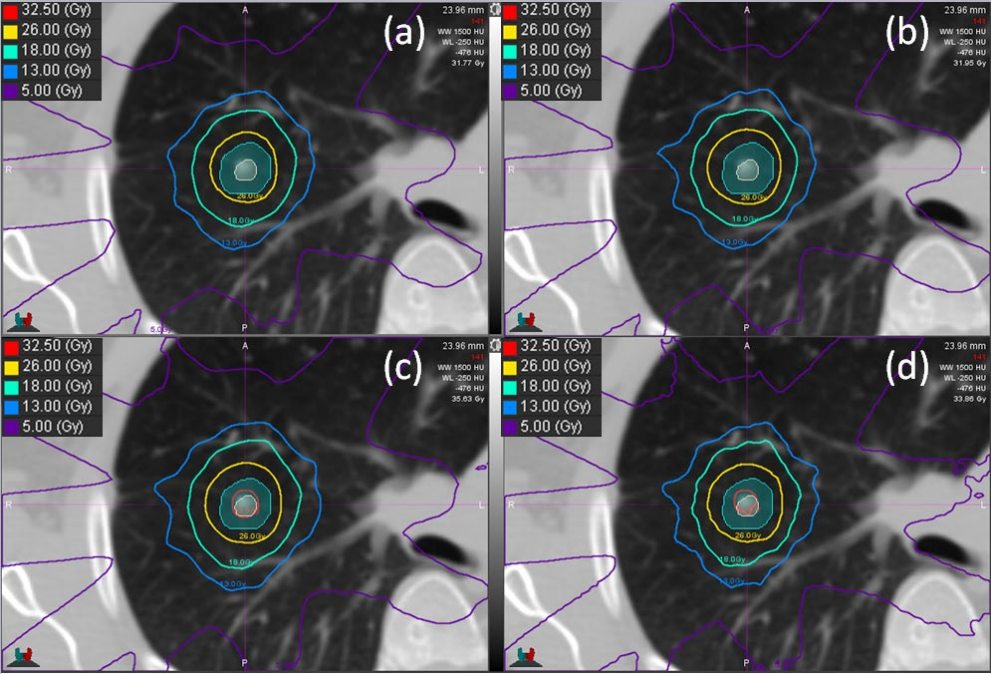
Isodose lines for (a) XiO, (b) AAA, (c) M3D, and (d) Monte Carlo for an example patient. The ITV (beige) and PTV (teal) are shown as colourwash. Note in the M3D and Monte Carlo images, the higher dose in the target (32.5 Gy isodose line) and the larger distance between the 26 Gy isodose line and the PTV contour.

Figure 1 shows a comparison of isodose lines from the four different calculation methods for a small lung lesion surrounded by lung tissue (Patient 6). There is a larger separation between the PTV contour and the 26 Gy isodose line in the Monte Carlo and M3D calculations compared with the AAA and XiO calculations. Higher central target doses were also typically observed with MC and M3D, shown by the appearance of the 32.5 Gy isodose line in the target.

**Figure 2:**
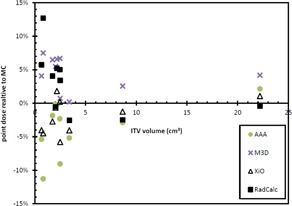
Point doses relative to Monte Carlo for each target at the calculation point in the center of the PTV.

The point doses calculated at the center of each of the ITVs for each of the algorithms is given in Fig. 2 and Table 2. The AAA algorithm point doses were lower than that calculated with MC in all but one case (Patient 8), with the maximum difference being −11.3% (p=0.016). There was no statistically significant difference between XiO and MC, with all points within 5.8%(p=0.059). The M3D doses were all greater than those calculated by MC, by up to 7.5%(p=0.005). The RadCalc‐calculated central ITV point doses are up to 13.1% higher than those calculated with MC (p=0.075). This is likely due to the inability of the RadCalc algorithm to take into account the lack of lateral scatter into the target when a small lesion is surrounded by lung tissue. A larger disagreement between all algorithms was observed with decreasing ITV size.

**Figure 3:**
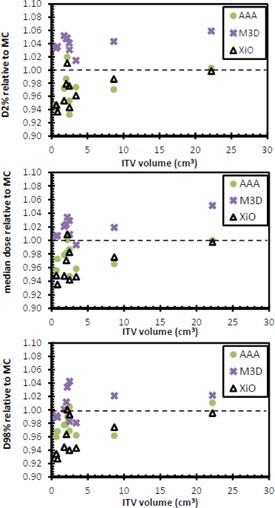
PTV D2% (top), median (middle), and D98% (bottom) values for each of the three calculation methods relative to Monte Carlo calculated dose.

Figure 3 and Table 3 show the PTV D2%, median, and D98% values for each calculation method compared with the MC calculation. The comparison is performed for Patient 6 for a unit density patient; the maximum difference between the algorithms was 1%. This suggests that the difference between the calculation models, independent of heterogeneity corrections, is at most 1%. The near minimum PTV doses were statistically significantly lower than MC when calculated with AAA and XiO (p=0.037 and p=0.007 for AAA and XiO, respectively). There was no statistically significant difference between near‐minimum PTV doses calculated with M3D compared with MC (p=0.075). AAA and XiO near‐maximum PTV doses were statistically significantly lower than MC over the population by up to 7% and 6%, respectively (p=0.016 and p=0.009 for AAA and XiO, respectively). The M3D algorithm‐calculated near‐maximum doses were greater thanthose calculated with MC, which was also statistically significantly over the population (p=0.005). The median PTV doses calculated with AAA and XiO were typically less than those calculated with MC (p=0.012 and p=0.009 for AAA and XiO, respectively). The M3D PTV median doses were greater than those calculated with MC (p=0.012).

**Table 2:** Point‐dose differences between each calculation algorithm and Monte Carlo.

*Patient*	*ITV (cc)*	*XiO*	*AAA*	*M3D*	*RC*
1	0.66	−4.0%	−5.4%	4.1%	5.7%
2	1.75	−2.7%	−1.8%	6.5%	4.1%
3	2.47	0.2%	−2.3%	6.7%	5.0%
4	2.50	−5.8%	−9.1%	0.7%	3.4%
5	2.05	−0.7%	−0.2%	5.5%	−0.5%
6	0.83	−4.5%	−11.3%	7.5%	12.7%
7	3.41	−4.1%	−5.2%	0.2%	−2.5%
8	22.2	1.1%	2.1%	4.2%	−0.4%
9	2.18	1.8%	−0.3%	6.6%	5.2%
10	8.66	−1.3%	−2.9%	2.6%	−2.5%
Average		−2.0%	−3.6% ^a^	4.4%^a^	3.0%
St. Dev.		2.6%	4.2%	2.6%	4.7%
p		0.059	0.016	0.005	0.075

^a^ Statistical significance.

**Table 3:** Near‐minimum, median, and near‐maximum PTV doses as compared with Monte Carlo calculation.

	*D98%*			*Median*			*D2%*		
*Patient*	*XiO*	*AAA*	*M3D*	*XiO*	*AAA*	*M3D*	*XiO*	*AAA*	*M3D*
6 (Hom.)	1.00	1.01	1.01	1.00	1.01	1.01	1.01	1.01	1.01
1	0.93	0.96	0.99	0.95	0.96	1.01	0.95	0.95	1.03
2	0.95	0.98	1.00	0.94	0.97	1.01	0.95	0.97	1.05
3	0.94	0.97	0.98	0.95	0.95	0.99	0.98	0.95	1.04
4	0.99	1.00	1.04	0.94	0.95	1.01	0.94	0.93	1.03
5	0.92	0.93	0.96	0.93	0.94	0.98	0.96	0.96	1.02
6	0.92	0.96	0.98	0.93	0.96	1.00	0.94	0.95	1.04
7	0.92	0.94	0.96	0.93	0.95	0.98	0.96	0.97	1.01
8	1.00	1.01	1.02	1.00	1.00	1.05	1.00	1.00	1.06
9	1.00	0.99	1.03	1.01	1.00	1.03	1.01	1.02	1.05
10	0.97	0.96	1.02	0.98	0.97	1.02	0.99	0.97	1.04
Average	0.95^a^	0.97^a^	1.00	0.96^a^	0.96^a^	1.01^a^	0.97^a^	0.97^a^	1.04^a^
St. Dev.	0.03	0.03	0.03	0.03	0.02	0.02	0.02	0.03	0.01
p	0.007^a^	0.037^a^	0.075	0.009^a^	0.012^a^	0.012^a^	0.009^a^	0.016^a^	0.005^a^

^a^ Statistically significant difference.

**Figure 4:**
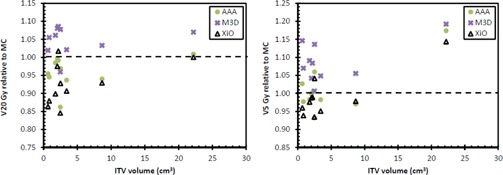
Ipsilateral lung V20 Gy (left) and V5 Gy (right) for each of the three calculation methods relative to Monte Carlo.

All original treatment plans (generated with the XiO TPS) satisfied the dose constraints specified in Table 1. The dose recalculated in each of the other calculation methods also satisfied all constraints given in Table 1. The AAA and XiO ipsilateral lung V20 Gy were lower than that calculated with MC (p=0.016 and p=0.015 for AAA and XiO, respectively) (see Fig. 4). The M3D V20 Gy was slightly higher than that calculated with MC (p=0.005). There was no statistically significant difference between AAA and XiO compared with MC for the ipsilateral lung V5 Gy; however, the V5 Gy for M3D was statistically significantly higher than for MC (p=0.005).

**Figure 5:**
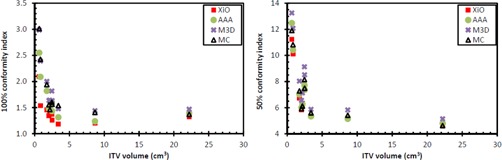
Conformity indices for 100% (left) and 50% (right) isodose lines.

The AAA and XiO calculations all resulted in lower CI 100% relative to MC (p=0.022 and p=0.005 for AAA and XiO, respectively) (see Fig. 5). The M3D CI 100% was higher than that calculated with MC (p=0.037). There was no statistically significant difference between AAA and MC for the CI 50%, but the XiO CI 50% was lower than that calculated with MC (p=0.022). The M3D CI 50% was greater than that calculated with MC (p=0.005). Greater CI values indicate the 100% and 50% isodose lines contained a larger volume than that calculated with XiO for the same number of monitor units.

The results of the M3D gamma analysis and RadCalc dose discrepancy are shown in Table 4. Pass rates were arbitrarily assigned for the M3D gamma pass criteria such that a ‘fail’ category was assigned if the M3D gamma pass rate for the 5%/1 mm criteria was <95%, or 3%/1 mm <80%. Per our institutional guidelines, a pass rate (total for all beams) of 5% was applied for the monitor unit check software (RadCalc).

**Table 4:** Mean target dose, 3D gamma passing rates from M3D calculation, and point‐dose calculation from RadCalc compared with the two planning system calculations. A pass (P) rate for 5%/1 mm was 95% and for 3%/1mm was 80%. A fail (F) for the point‐dose verification was >5%.

	*M3D Relative to XiO*			*M3D Relative to Eclipse*			*RadCalc Relative to XiO*		*RadCalc Relative to Eclipse*	
*Patient*	*5%/ 1 mm*	*3%/ 1 mm*	*P/F*	*5%/ 1 mm*	*3%/ 1 mm*	*P/F*	*Point Dose*	*P/F*	*Point Dose*	*P/Fail*
1	96.9%	84.7%	P	98.4%	92.1%	P	‐9.3%	F	11.8%	F
2	92.9%	74.1%	F	97.4%	87.2%	P	−6.5%	F	6.0%	F
3	97.8%	83.2%	P	97.2%	87.4%	P	−4.1%	P	7.5%	F
4	98.7%	89.1%	P	98.8%	91.0%	P	−8.3%	F	13.7%	F
5	95.6%	81.8%	P	98.7%	93.5%	P	0.0%	P	−0.3%	P
6	97.0%	85.2%	P	98.6%	89.5%	P	−15.3%	F	27.0%	F
7	98.1%	89.0%	P	99.4%	95.2%	P	−1.2%	P	2.8%	P
8	98.8%	89.8%	P	98.9%	91.6%	P	1.6%	P	−2.5%	P
9	99.3%	90.1%	P	97.7%	90.2%	P	−2.6%	P	5.5%	F
10	98.3%	84.6%	P	98.0%	91.2%	P	−0.3%	P	0.4%	P

## IV. DISCUSSION

**Figure 6:**
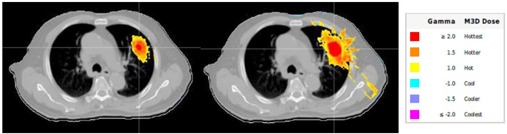
The gamma distribution map comparing M3D with XiO (left) and Eclipse (right) for Patient 2 at 5%/1 mm

Treatment plans are typically assessed by the referring clinician based on a number of criteria and dose parameters including dose constraints to OARs and dose distribution parameters to the PTV such as those recommended by ICRU 83. The role of the physicist is to understand the limitations of the TPS, identify any errors that may be due to the local implementation of the TPS dose model, and confirm the ability of the linac to deliver the planned dose. Delivery of the treatment plan to a phantom may verify the latter; however, as it is impractical to generate a phantom that mimics individual patient geometry, including tissue heterogeneities, measurements made in a phantom are not sufficient to verify the dose distribution in a patient. We therefore assessed the performance of commercial 3D plan review software, M3D, which utilizes a Type B algorithm, and compared this with a commonly used monitor unit check program, RadCalc, that uses a simplistic approach to modeling lateral scatter. While RadCalc is used for comparison of point dose verification, M3D provides a method to verify 3D dose distributions, including within target volumes and OARs. Using the recommended ICRU 83 dose reporting parameters, there were clearly significant differences (>5%) between the M3D and TPS (XiO and Eclipse AAA) calculations that would be difficult to resolve by direct measurement for the reasons previously discussed. Furthermore, we identified instances of discrepancy between M3D and our reference MC calculations, suggesting the M3D calculations also have limitations when used for reporting these parameters. The value of M3D in the scenario we present here (lung SBRT), therefore, appears to be in the evaluation of multiple points through the treatment volume (through a gamma analysis) and review of the DVH data which can be assessed in terms of the plan achieving the planning objectives — primarily achieving the minimum peripheral (ablative) dose and achieving sharp dose falloff outside of the target. There is clearly still a requirement to assess the spatial distribution of points that fail the gamma criteria to identify the reasons why these points failed and determine if the discrepancies are clinically relevant. In the cases investigated, all but 1 plan passed the 5%/1 mm <95% or 3%/1 mm <80% criteria, and the points that failed this criteria are indicated in Fig. 6. In contrast, four and six of the 10 patient plans failed the 5% criteria for the point‐dose verification using RadCalc for the XiO and Eclipse plans, respectively. While there are clear limitations in both the M3D and RadCalc algorithms, the ability to verify clinically relevant plan parameters is highly beneficial.

The number of cases included in our study is too small to recommend gamma pass rates for the criteria we used in our study. Furthermore, we were unable to perform a gamma analysis comparing M3D and MC and, therefore, unable to report the accuracy of M3D using the gamma analysis criteria. We have demonstrated how the results of the point dose and ICRU 83 parameter calculations provide a useful indicator of the uncertainties in the M3D calculations. M3D‐calculated dose in the context of lung SBRT appears to be similar to Monte Carlo‐calculated doses, with an increase in predicted target dose observed with M3D and Monte Carlo relative to XiO and AAA calculated doses. Our small study of 10 patents indicated the relative increase in target dose increases with decreasing target size. Previous studies have shown conflicting results to that found in the current study. Panettieri et al.^(15)^ compared Monte Carlo with the Pinnacle^3^ CCC algorithm (Philips Radiation Oncology Systems, Fitchburg, WI) for small lung targets and found slightly higher near‐minimum PTV doses with Monte Carlo compared to CCC, but very little difference in the near‐maximum PTV doses between the two algorithms. Vanderstraeten et al.^(16)^ compared two convolution/superposition algorithms (Pinnacle^3^ and Helax‐TMS (MDS Nordian, Ottawa, ON, Canada) with Monte Carlo for IMRT lung tumors, showing Monte Carlo‐calculated maximum PTV doses higher than those calculated with convolution/superposition. Increased target and lung doses were observed when Monte Carlo calculation in the Monaco treatment planning system was compared to the CCC algorithm in XiO,^(17)^ which is in agreement with our results. The M3D CCC is based on the Pinnacle^3^ CCC algorithm, but with fewer approximations in the implementation due to the use of a GPU.^(6)^ The current results, however, suggest that for small lung targets, the M3D‐calculated PTV D2% was up to 6% higher than with Monte Carlo, whilst maintaining similar median and D98% doses. The increased doses observed M3D also manifest as increased lung V20 Gy and V5 Gy and subsequent conformity indices. The increases in the lung dose, although relatively high, represent a small physical volume increase due to the small targets analyzed in this study.

The disparity between the calculation techniques for point doses in the center of the target was stark; this highlights the limitations of plan verification with different algorithms based on a single point. There were still significant differences in the volumetric‐dose metrics of M3D and Monte Carlo compared with the treatment planning system calculations, of up to 7% for the near‐maximum target dose. In the context of lung SBRT, validation of the minimum peripheral dose is paramount, as well as the dose to adjacent surrounding structures. In light of this, one may expect M3D to be in closer agreement to Monte Carlo when evaluating coverage (D98% in the current study), but still up to 8% different to the treatment planning system calculation. Thus the results suggest a similar challenge to that presented by point dose validation may be faced when using M3D to validate plans calculated with superposition algorithm in XiO and to a lesser extent AAA in Eclipse. When performing a plan check using M3D, the results suggest that one will be exceeding the planned target dose and increasing the dose to adjacent normal tissue. The former is not necessarily problematic in lung SBRT. However, if the target is particularly close to critical structures that are close to tolerance dose in the original treatment plan, this may present a clinical dilemma if tight plan check tolerances are used.

Target coverage and dose to OARs met the planning objectives according to the TPS, Monte Carlo, and Mobius calculations, thus the outcome of these calculations would not have impacted on the decision to treat. However, the disparity in results between each of the dose calculation methods highlights a particular challenge in interpreting the results of clinical trials. In the case of different centers using different planning systems, each with a different algorithm, the reported dose to the target and OARs may not be directly comparable, leading to uncertainties in defining tumor and OAR dose response metrics.^(18)^

## V. CONCLUSIONS

The dose calculation in a commercial plan checking software which uses a convolution/superposition dose calculation algorithm has been compared with two treatment planning system calculations and Monte Carlo for lung SBRT treatment planning. We conclude that M3D is a useful tool for 3D assessment of lung SBRT plans created with the commercial TPSs considered in this report. There remain some significant discrepancies (>5%) in comparing single point doses and dosimetry parameters recommended by ICRU 83 when compared with the reference dataset generated by MC. The M3D user, therefore, needs to understand the limitations of M3D in predicting dose in using the ICRU 83 dosimetric parameters but appreciate the M3D benefits when considering verification by direct measurement typically excludes verification of dose distribution in the presence of significant tissue heterogeneities encountered in typical lung SBRT.

## ACKNOWLEDGMENTS

The authors would like to acknowledge Mobius Medical Systems for useful discussion of the Mobius dose calculation algorithm. The authors would like to also acknowledge Ajit Mullen and Elena Ungureanu for assistance with data collection. The author (B.M.O) acknowledges funding from NHMRC Program Grant No. 1036078, ARC Discovery Grant No. DP120100821.

## COPYRIGHT

This work is licensed under a Creative Commons Attribution 3.0 Unported License.
